# Do adverse events after manual therapy for back and/or neck pain have an impact on the chance to recover? A cohort study

**DOI:** 10.1186/s12998-019-0248-9

**Published:** 2019-06-12

**Authors:** Vesa Tabell, Ina M. Tarkka, Lena W. Holm, Eva Skillgate

**Affiliations:** 1 Faculty of Sport and Health Sciences, University of Jyväskylä, FI-40014 Jyväskylä, Finland; 2 Musculoskeletal & Sports Injury Epidemiology Center, Institute of Environmental Medicine, Karolinska Institutet, Box 210, SE-17177 Stockholm, Sweden; 3 Sophiahemmet University, Box 5605, SE-11485 Stockholm, Sweden; 4 Naprapathögskolan - Scandinavian College of Naprapathic Manual Medicine, Kräftriket 23A, SE-11419 Stockholm, Sweden

**Keywords:** Manual therapy, Adverse event, Low back pain, Neck pain, Recovery

## Abstract

**Background:**

Manual therapy is a commonly used treatment for patients with back and neck pain. Studies have shown that manual therapy-related adverse events are mainly short in duration and mild or moderate by their intensity, affecting up to 50% of the patients. If the presence of adverse events has an impact on the chance to recover from back/neck pain is poorly understood. The aim of this study was to investigate if mild or moderate adverse events after manual therapy has an impact on the chance to recover from back/neck pain in men and women.

**Methods:**

A prospective cohort study of 771 patients with at least three treatment sessions in a randomized controlled trial performed in January 2010 – December 2013. Adverse events within 24 h after each treatment were measured with questionnaires and categorized as: no, mild or moderate, based on bothersomeness. Outcome measure was the perceived recovery at seven weeks and at three months follow-up. Odds Ratios (OR) and 95% confidence intervals (CI) were calculated by Logistic regression to investigate the associations between the exposure and outcome, and to test and adjust for potential confounding.

**Results:**

There were no statistically significant associations observed between the experience of mild or moderate adverse events and being recovered at the seven weeks follow-up. The only statistically significant association observed at the three months follow-up was for mild adverse events in men with an OR of 2.44, 95% CI: 1.24–4.80 in comparison to men with no adverse events.

**Conclusion:**

This study indicates that mild adverse events after manual therapy may be related to a better chance to recover in men.

**Trial registration:**

The study is based on data from a trial registered in Current Controlled Trials (ISRCTN92249294).

## Background

Patients suffering from disability related to low back pain (LBP) and neck pain (NP) have multiple evidence-based treatment options to choose from. Manual therapy (MT) seems to be a treatment option as effective as other modalities [1] and a cost-effective [2] treatment for certain musculoskeletal disorders. MT provided by naprapaths is well established in Sweden, Finland and Norway and has been found to be an effective and cost-effective treatment [3–5].

Studies have shown that MT related adverse events (AE) are mainly short in duration and mild or moderate by their intensity, affecting up to 50% of the patients [6–8]. AE after spinal MT is reported as self-limiting, transient and located in the musculoskeletal system [9], usually including symptoms like musculoskeletal pain, tiredness, stiffness, dizziness, radiating discomfort, headache or nausea [10]. AE are reported more commonly by female patients [8–10].

It is generally accepted to measure AE in terms of severity, duration and nature [8, 11, 12], and to classify AE as: mild, moderate or major/intense [11, 12]. Severe, irreversible AE are excluded from this classification. Most negative treatment-related AE is defined as pain and loss of function with impact on daily living or work [12, 13].

We have previously [8] shown that AE in patients with LBP and/or NP treated with naprapathic MT did not differ between MT with or without spinal manipulation. MT provided by naprapaths to treat pain and pain related disability in the musculoskeletal system, is a combination of manual techniques as spinal manipulation/mobilization, stretching and massage. A systematic review of Carnes et al. [7] states that MT interventions produced more minor or moderate AE than general practitioner care, and equal number of AE as exercise therapy and less AE than drug therapy.

The role of AE for the positive effect of MT on LBP and NP has been studied by several researchers. Some studies have shown that AEs are not associated with recovery at three months follow-up for NP patients [14–16]. In contrary, others suggest that patient perception of AE being present may be of importance for a positive effect of MT [13]. The relationship between AE after MT for other conditions than NP, and if the potential association is modified by sex is still not well known. Our hypothesis is that AE after MT influence the prognosis in the short term, and that the occurence of AEs may be related to treatment induced cascade of neurophysiological responses [17] of the symptomatic tissues. This proprioceptive information including pain, [18] could be temporary and is induced by biomechanical changes in tissue loading. The potential influence of AE on the prognosis may be mediated through cultural and psychological characteristics e.g. expectations, and may differ between men and women.

The aim of this study was to study if mild or moderate AE after naprapathic MT have an impact on the chance to recover from back and/or neck pain in men and women.

## Methods

This study is a secondary analysis of data from a randomized controlled trial (RCT) performed in January 2010 – December 2013, in Stockholm, Sweden. The study design is a prospective cohort study.

### Study population

The study participants were selected among the included in the RCT called the Stockholm Manual Intervention Trial (*n* = 1057), with the main aim to compare the treatment effect and the risk of AEs between three different combinations of MT (spinal manipulation, mobilization, muscle stretching and massage) for patients seeking care for non-specific LBP and/or NP [8]. The study participants were patients (18–65 years old) seeking care for back and/or neck pain at the education clinic of the Scandinavian College of Naprapathic Manual Medicine in Stockholm, Sweden. Students in their seventh semester of the education delivered the treatments. Details of the trial including inclusion and exclusion criteria is presented elsewhere [8].

The study population in the present study was 771 patients. The inclusion criteria was to have had at least three treatment sessions and to have answered questionnaires about AE after the first three visits.

### Exposure

AEs after MT, measured by paper questionnaires at all return visits at the clinic, were the exposure in this study. The questionnaire was given to the patients to fill in while waiting for the therapist and was handed in before the treatment session started. If the third treatment was the last session, a research assistant contacted the patient by telephone within a week to collect information regarding potential AE after the third treatment. Each of the AE questionnaire included eight questions concerning any AE present within 24 h after treatment. The introduction text on the questionnaire was*; It happens that patients experience adverse events in connection with manual treatment therapy. Therefore, we wonder if you experienced any of the following events. Note that only symptoms within 24 h debut after the treatment session shall be reported.* AE for the patients to report on were 1. Tiredness, 2. Soreness in muscles, 3. Stiffness, 4. Increased pain, 5. Nausea, 6. Headache, 7. Dizziness or 8. “Other”. Bothersomeness from AE was measured with a numerical rating scale (NRS) from 0 to 10 (0 = had not bothered them at all, and 10 = had bothered them in the worst possible way). If patient had not experienced AE, bothersomeness was classified as 0. We took the highest NRS-value for bothersomeness from the eight possible AE from each of the three return visits. These values were used to calculate the mean of bothersomeness of the three sessions for each patient. The exposure AE was then categorized into three levels based on the mean score, as no (< 1), mild (1–3) or moderate/major (≥4). A low proportion of patients (3%) reached up to NRS > 7 (major AE), meaning that this group was to small to be analyzed separately.

Since many patients only needed three treatment sessions, we included only information from the AE-questionnaires delivered after the first three visits, so that all study participants had had the same number of treatments and thereby theoretically the same risk of AE after treatment.

### Outcome

**Fig. 1:**
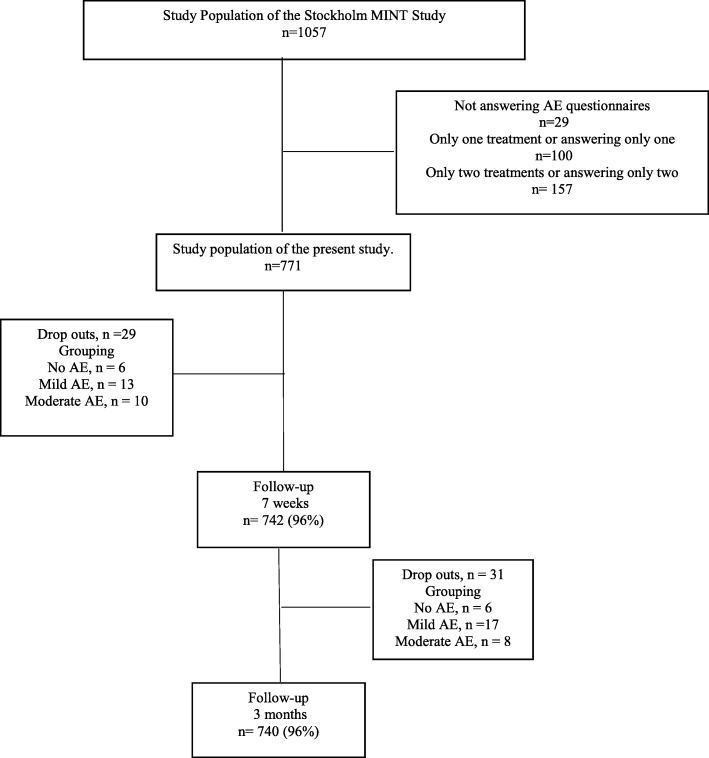
Flowchart of the inclusion process

The outcome perceived recovery was self-reported measured with follow-up questionnaires at seven weeks and three months by the Global Perceived Recovery Question (6-point Likert scale). The question used was; “*Which of the following statements best matches how you feel your symptoms in the neck/back have changed since you joined the study*”. The answering options were; a. “Feel no pain at all and no other symptoms from my neck or/and back”, b. “Is considerably better”, c. “Is slightly better”, d. “No Improvement”, e. “Is slightly worse”, f. “Is considerably worse”. Answers a and b were defined as recovered, and the rest as not recovered. Similar definitions and 6-point rating scale has been used in several studies [8, 15]. The loss to follow-up of the outcome measured by questionnaires was 4% at seven weeks as well as at three months (Fig. 1).

### Potential confounders

Potential confounding factors for the associations between the exposure and the outcome was identified through theoretical and empirical considerations and based on data available from the baseline questionnaire were the factors reported in Table 1.

**Table 1:** Baseline characteristics of study participants stratified by AE after first three visits, mean (*n* = 771)

	All		No AE (NRS < 1) (*n* = 182, 24%)		Mild AE (NRS 1–3) (*n* = 401, 52%)		Moderate AE (NRS ≥4) (*n* = 188, 24%)	
Characteristics	No.	%	No.	%	No.	%	No.	%
Mean age (SD)	36 (12)		36 (12)		36 (12)		36 (12)	
Gender								
Women	545	71	106	58	285	71	154	82
Men	226	29	76	42	116	29	34	18
Painful area								
Back	255	33	79	43	123	31	53	28
Neck	422	55	83	46	219	55	120	64
Back/Neck	94	12	20	11	59	15	15	8
Duration of pain								
Acute/subacute (≤3 months)	488	63	113	62	260	65	115	61
Chronic (> 3 months)	283	37	69	32	141	35	73	39
Previous similar complaints	599	78	137	75	312	78	150	80
Pain at baseline (SD)^a^	771		5 (2)		5 (2)		6 (2)	
Disability at baseline (SD)^b^	771		2 (2)		2 (2)		3 (2)	
Recovery expectations (SD)^c^	771		6 (3)		6 (3)		6 (3)	
General health								
Good or better	731	95	173	95	379	95	179	95
Fair	40	5	9	5	22	6	8	4.5
Poor	1	< 1	0	0	0	0	1	0.5
Education								
Elementary school 1–9 y	26	3	8	4	10	3	8	4
High school 10–12 y	268	35	70	39	128	32	70	37
University 13–15 y	375	49	80	44	205	51	59	48
Higher academic education ≥16 y	102	13	24	13	58	15	20	11
Distress (SD)^d^			5 (1)		5 (1)		5 (1)	
Daily smoking	105	14	18	10	56	14	31	17
RCT group								
NMT	244	32	62	34	121	30	61	32
NMT- no manipulation	265	34	53	29	148	37	64	34
NMT- no stretching	262	34	67	37	132	33	63	34

Abbreviations: *AE* adverse events, *NRS*, number rating scale, *SD* standard deviation, *RCT* randomized controlled trial, *NMT* Naprapathic manual therapy

^a^Average of three questions from (0 = no pain and 10 = worse possible pain) how strong is the pain right now, intensity of the worst pain in the last four weeks and average of the last four weeks

^b^Average of three questions from (0 = had no effect on function and 10 = impossible to perform activities) affection to function in the last four weeks, affection to social activities and affection to work

^c^Expectation of asymptomatic pain area in seven weeks (0 = not at all and 10 = very likely)

^d^ Have you felt gloomy and sad in the last four weeks (1 = all the time and 6 = not at all)

### Statistical methods

Descriptive statistics were used to summarize baseline characteristics. Several baseline variables were dichotomized and categorized for analyses. Duration of pain was dichotomized to acute/subacute (≤3 months) and chronic (> 3 months). Pain intensity at baseline was measured with three numerical rating scales (NRS 0–10); pain right now, worst pain in the last four weeks and average pain of the last four weeks. Pain intensity was operationalized as the total mean value of the three scales. Pain related disability was operationalized and calculated in the same way but based on the following three questions about:1. how pain has hindered daily activities in the last four weeks, 2. how much pain has affected social activities in the last four weeks and 3. how much the pain has interfered with work during the last four weeks (NRS; 0 = had no effect to function and 10 = impossible to perform activities). General health categorization (1. Excellent, 2. Very good, 3. Good, 4. Fair and 5. Poor) was categorized as “good or better” (1, 2 and 3), “fair” (4) and “poor” (5).

To control the confounders statistically we used comparison of odds ratios calculated with logistic regression. Each potential confounder was tested by adding it to the model one at a time. If the adjusted model changed the point estimate with 10% or more, confounding was considered present and the factor was added to the final model. The test for confounding was performed for the total study sample as well as for men and women separately. The variable “treatment arm” of the original RCT was included in all the models no matter if it was a confounder or not.

Recovery expectations were defined as the patients self-rated likelihood of being symptom-free after seven weeks on an NRS 0–10 (0 = not at all likely and 10 = very likely). The variable was used as a continuous variable in the analyses. Distress was defined by asking to which extent of time the patient felt gloomy and sad in the last four weeks, conducted on an NRS 1–6 (1 = all the time and 6 = not at all), where all six categories were used in the analyses.

The sample size provided a power of 80% to find a relative risk of 1,3 for the outcome between the exposed and unexposed (epinet.se). Logistic regression modelling was used to determine odds ratios (OR) and 95% confidence intervals (CI 95%) for AE effect on perceived recovery. Statistical analysis was performed using IBM SPSS version 22 for Mac.

## Results

Patient characteristics at baseline stratified by bothersomeness of AEs are shown in Table 1. The mean age of all was 36 years and 71% were women. Chronic pain was reported by 37% of the patients, the average pain was five (NRS 0–10) and the average of recovery expectations was six (NRS 0–10). General health was reported to be at least good by 95% of patients. AE were commonly reported; 81% of women and 66% of men reported some AE. No severe irreversible AE were reported.

The associations between AE and the chance to recover at seven weeks and three months follow-ups as well as the proportion of recovered patients in their respective groups are presented in Tables 2 and 3. There were no statistically significant associations observed between the experience of mild or moderate AE and being recovered at the seven weeks follow-up. The only statistically significant association observed at the three months follow-up was for mild AE in men with an OR of 2.44, 95% CI: 1.24–4.80 in comparison to men with no AE.

**Table 2:** Associations between AE and recovery at seven weeks’ follow-up presented as crude and adjusted Odds Ratio (OR) with 95% confidence intervals (95% CI) (*n* = 742)

	All OR (95% CI)			Men OR (95% CI)			Women OR (95% CI)	
AE	N^a^ (n^b^)	Crude^c^	Adjusted^d^	N^a^ (n^b^)	Crude^c^	Adjusted^d^	N^a^ (n^b^)	Crude^c^
No (NRS < 1)	176 (94)	1.0 (ref.)	1.0 (ref.)	74 (35)	1.0 (ref.)	1.0 (ref.)	102 (59)	1.0 (ref.)
Mild (NRS 1–3)	388 (212)	1.05 (0.74–1.50)	1.14 (0.79–1.64)	111 (61)	1.36 (0.76–2.46)	1.46(0.78–2.69)	277 (151)	0.88 (0.56–1.39)
Moderate/major (NRS ≥ 4)	178 (86)	0.82 (0.54–1.24)	0.92 (0.60–1.41)	32 (16)	1.13 (0.49–2.59)	1.25 (0.53–2.93)	146 (70)	0.68 (0.41–1.14)

Abbreviations: AE, adverse events; OR, Odds Ratio; CI, confidence interval; NRS, numeric rating scale(0–10)

^a^Number of patient in AE group

^b^Number of recovered patient measured by perceived recovery in AE group

^c^Treatment arm included in analysis

^d^Adjusted for treatment arm and recovery expectations

**Table 3:** Associations between AEs and recovery at three months’ follow-up presented as crude and adjusted Odds Ratios (OR) with 95% confidence intervals (95% CI) (*n* = 740)

	All OR (95% CI)			Men OR (95% CI)			Women OR (95% CI)		
Adverse events	N^a^(n^b^)	Crude^c^	Adjusted^d^	N^a^ (n^b^)	Crude^c^	Adjusted^e, f^	N^a^(n^b^)	Crude^c^	Adjusted^g^
No (NRS < 1)	176 (93)	1.0 (ref.)	1.0 (ref.)	73 (34)	1.0 (ref.)	1.0 (ref.)	103 (59)	1.0 (ref.)	1.0 (ref.)
Mild (NRS 1–3)	384 (210)	1.08 (0.75–1.54)	1.19 (0.82–1.72)	112 (67)	1.72 (0.95–1.12)	2.44 (1.24–4.80)	272 (143)	0.83 (0.53–1.91)	0.84 (0.55–1.40)
Moderate/major (NRS ≥ 4)	180 (77)	0.67 (0.44–1.01)	0.76 (0.50–1.18)	33 (12)	0.66 (0.28–1.55)	0.58 (0.22–1.52)	147 (65)	0.60 (0.34–0.99)	0.68 (0.40–1.13)

Abbreviations: AE, adverse events; OR, Odds Ratio; CI, confidence interval; NRS, numeric rating scale (0–10)

^a^Number of patient in AE group

^b^Number of recovered patient measured by perceived recovery in AE group

^c^Treatment arm included in analysis

^d^Adjusted for treatment arm and recovery expectations

^e^Mild AE; Adjusted for treatment arm, recovery expectations and pain location

^f^Moderate AE; Adjusted for treatment arm and recovery expectations, distress, disability at baseline and pain duration

^g^Adjusted for treatment arm and pain at baseline (confounder for women regarding moderate AE)

## Discussion

The result of this secondary analysis of data from a large RCT suggests that mild AE after MT may improve the chance to be recovered three months after treatment in men seeking care for non-specific LBP and/or NP.

AE was not a prognostic factor when both genders were analyzed together. This is consistent with results in previous studies, where AE after MT were unrelated to outcome after three months [14–16]. Our results show that AE are common and that most cases are mild, which are in line with the results in previous studies [6, 7, 9].

### Strengths and limitations

Important strengths of this study were the large study population, the careful management of confounders and the high response rate. Potential confounders were identified through theoretical and empirical considerations and were available from the extensive baseline questionnaire. Recovery expectation is a well-known prognostic factor for NP and LBP and was a confounder adjusted for in most of the final analyses [19, 20]. All study participants were patients seeking care for their complaints, thus they may have higher expectations for recovery than persons who do not seek care. However, this is not a source of bias since the study is etiological and recovery is compared between groups. A limitation is that there could be residual and unmeasured confounding bias of the results, caused by use of medication and from sport or work related traumas or overloads. Further patho-anatomical, neuro-physiological and cognitive-behavioral factors may effect recovery and thus potentially confound the associations.

We used the outcome perceived recovery that is considered to increase the external validity of the results [21] and that is a reliable assessment of current health status in people with musculoskeletal disorders [22]. Perceived recovery seems to correlate with changes in pain and disability scores during MT [23].

Even though data from long-term follow-ups is available in the original trial on which this study is based, we decided not to investigate the long-term effect of AE on recovery. This decision was made based on our hypothesis that it would be improbable that a short time reaction after a treatment would impact the result of the treatment in the long term.

The questionnaire used to measure the exposure AE and the patients rating of bothersomeness from AE has not been formally tested with regard to validity and reliability. This means that there may be a risk for non-differential misclassification of exposure, which may dilute the associations studied**.** Since the intervention strategy was to give six treatments within six weeks, the absolute majority of the AE questionnaires were filled in within a week after the treatment session. This means that the risk of misclassification of exposure due to long recall periods is low. AE were categorized into nonexistent, mild or moderate, demonstrating the concept of Carnes et al. [11] with the same NRS values as a previous study [24]. Bothersome AEs (NRS > 7) were also considered by Carnes et al. [11]. However, only a very low proportion of patients (3%) reached up to this level in our study sample thus the group was too small to study separately. These were included in the category “moderate” in the analyses. Further, choosing the highest value from each of the three questionnaires and calculating the mean of the three sessions does not take into account the potential cumulative effect of multiple AE in a single session. This may constitute a limitation in the classification of the exposure. Furthermore, studying the first three sessions combined do not determine if there is a specific effect on any or some of the individual sessions.

The result of this study adds to the knowledge that recovery from pain is a complex concept. Some tissues like intervertebral discs and ligaments, compared to muscles, responds slowly and perhaps incompletely to biomechanical chances [25]. After biomechanical unloading procedures achieved by MT and conditioning, the response could also be slow. Using MT and/or exercise produces forces interaction between motor and sensory control of the entire spine and related joints. This affects load-sensitive nerve endings located in muscle and tendons providing proprioceptive information including pain [18]. An alteration in loading of spine from “pain state” to “relief” needs readjustments in sensory-motor control and environment around sensory nerves [18]. These processes may have a role in the occurrence of AEs. Potential effect mechanisms may not only be biomechanical, but related to context and expectations. This may explain that we did find statistically significant associations in men but not in women.

Treatment related AE may be considered as a response to a biomechanical adaptation where nociceptors are stimulated through the unloading of painful tissues. Emphasis shall however be on recognizing unwanted severe changes so that they can be avoided. Since the presence of mild and moderate AE don’t improve the chance to recover in women, AE should be avoided especially in women.

## Conclusion

This study indicates that mild adverse events after manual therapy may be related to a better chance to recover in men.

## Abbreviations

AE: Adverse Events
CI: Confidence Interval
LBP: Low Back Pain
MT: Manual Therapy
NP: Neck Pain
NRS: Numerical Rating Scale
OR: Odds Ratio
RCT: Randomized Controlled Trial
